# Semaglutide *vs*. liraglutide and incidence of diabetes and cardiovascular disease: A target trial emulation using real‐world data

**DOI:** 10.1002/bcp.70711

**Published:** 2026-07-16

**Authors:** Ethan J. Cannon, Wendy Wang, Faye L. Norby, Rob F. Walker, Shalini Kulasingam, Lisa S. Chow, Joel F. Farley, Pamela L. Lutsey

**Affiliations:** ^1^ Division of Epidemiology & Community Health University of Minnesota School of Public Health Minneapolis Minnesota USA; ^2^ Department of Epidemiology, Peter O'Donnell Jr. School of Public Health UT Southwestern Medical Center Dallas Texas USA; ^3^ Department of Internal Medicine UT Southwestern Medical Center Dallas Texas USA; ^4^ Department of Tropical Medicine and Infectious Diseases Tulane University Celia Weatherhead School of Public Health New Orleans Louisiana USA; ^5^ Department of Epidemiology Tulane University Celia Weatherhead School of Public Health New Orleans Louisiana USA; ^6^ Division of Diabetes, Endocrinology and Metabolism, Department of Medicine University of Minnesota Medical School Minneapolis Minnesota USA; ^7^ Department of Pharmaceutical Care & Health Systems University of Minnesota College of Pharmacy Minneapolis Minnesota USA

**Keywords:** cardiology, cardiovascular, diabetes, endocrinology, epidemiology, nutrition, obesity, pharmacoepidemiology, public health

## Abstract

**Aims:**

The glucagon‐like peptide‐1 receptor agonists (GLP‐1 RAs) semaglutide and liraglutide are approved for chronic weight management. Both drugs also have favourable effects on the risk of developing diabetes and cardiovascular disease (CVD) compared with placebo, but their comparative effectiveness for these outcomes is unclear.

**Methods:**

We employed an active comparator, new user design to emulate a target trial using data from MarketScan claims databases. Enrolees free of diabetes and CVD prescribed semaglutide or liraglutide during 2021–2023 were matched 1:1. Primary endpoints included incident diabetes and CVD (myocardial infarction, heart failure or stroke). We used Cox regression adjusted for a propensity score reflective of comorbidities and medication use.

**Results:**

We matched 57 456 GLP‐1 RA users (mean age 45; 83% female). Over 1‐year mean follow‐up, we observed 1104 diabetes events and 57 CVD events. After regression adjustment, semaglutide users had 12% lower risk of diabetes (hazard ratio [HR]: 0.88; 95% CI: 0.78, 0.99). For this endpoint, the proportional hazards assumption was violated (*p* < .0001), so results were stratified by follow‐up time. During the first 6 months of follow‐up, semaglutide use was not associated with risk of diabetes compared with liraglutide use (HR: 0.99 [0.82–1.18]), but a significant association was present thereafter (HR: 0.80 [0.68, 0.94]). We did not detect a difference in risk of CVD events (HR: 1.25 [0.74–2.11]).

**Conclusions:**

In this real‐world study, semaglutide users had lower risk of incident diabetes compared with liraglutide users. Additional research is needed to elucidate comparative effectiveness of these drugs for cardiovascular risk.

**What is already known about this subject**

- Semaglutide and liraglutide each reduce weight, lower HbA1C and decrease the risk of major adverse cardiovascular events compared with placebo.
- Current evidence indicates that semaglutide causes greater weight and HbA1C reduction compared with liraglutide.
- The comparative effectiveness of these drugs for the prevention of incident diabetes and cardiovascular disease is unknown.

**What this study adds**

- In patients without diabetes using GLP‐1 RAs for weight management, semaglutide use was associated with a lower risk of incident diabetes compared with liraglutide use.
- The effect was more apparent later during follow‐up, in alignment with the findings of clinical trials that showed semaglutide had a slower but ultimately stronger impact on other endpoints.

## 1 INTRODUCTION

Semaglutide and liraglutide are glucagon‐like peptide‐1 receptor agonists (GLP‐1 RAs) initially developed to treat diabetes. Functions of this drug class include stimulating insulin secretion, inhibiting glucagon secretion, delaying gastric emptying and promoting satiety.^1^ As evidence of benefit in other conditions has accrued, the US Food and Drug Administration (FDA) has approved additional indications for certain GLP‐1 RAs, including chronic weight management for daily liraglutide (maintenance dose 3.0 mg^2^) in December 2014 and weekly semaglutide (maintenance dose 2.4 mg^3^) in June 2021.^1^

Obesity affects 40% of US adults^4^ and is a leading risk factor for both diabetes and cardiovascular disease (CVD). Therefore, it is imperative to understand the impacts of semaglutide and liraglutide on the development of these conditions, as prescriptions for GLP‐1 RAs are increasing dramatically.^5^ Clinical trials have provided important evidence on this topic: In patients with preexisting CVD but without diabetes enrolled in the SELECT trial, semaglutide reduced the risk of incident diabetes^6^ and cardiovascular events^7^ compared with placebo. Likewise, the placebo‐controlled SCALE trial found that liraglutide significantly lowered the risk of incident diabetes.^8^ Moreover, patients with diabetes and high cardiovascular risk who received a lower dose version of liraglutide had a reduced incidence of cardiovascular events compared with placebo in the LEADER trial.^9^ Some head‐to‐head clinical trials comparing varying doses of semaglutide and liraglutide have been conducted in different patient populations, and results of these studies have generally favoured semaglutide for outcomes such as HbA1C and bodyweight reduction.^10^, ^11^, ^12^

Despite these important findings, key knowledge gaps remain. First, prior research has not directly addressed the comparative effectiveness of semaglutide *vs*. liraglutide in relation to incident diabetes. Second, no head‐to‐head trial assessing risk of CVD has been conducted for these drugs. Third, prior evidence on these outcomes has originated primarily from studies composed of patients with established CVD, which fails to represent a large segment of the overall population of semaglutide and liraglutide users. Addressing these questions with future randomized clinical trials would be resource‐intensive and time‐consuming. Moreover, observational research can provide perspective on the use of these drugs under real‐world conditions.^13^

Therefore, in the present study, we approached this topic by emulating a clinical trial using data from deidentified administrative claims databases. Using a target trial emulation framework,^14^, ^15^ we performed a head‐to‐head comparison of semaglutide and liraglutide, assessing endpoints of incident diabetes and CVD. As prior evidence has indicated a stronger effect of semaglutide on HbA1C and weight reduction,^10^, ^11^, ^12^ we hypothesized that compared with liraglutide, semaglutide use is associated with lower risk of both diabetes and CVD.

## 2 MATERIALS AND METHODS

### 2.1 Data source

We leveraged data from the Merative™ MarketScan® Commercial and Medicare Research Databases for the years 2018–2023. These deidentified administrative claims databases include enrolment records along with inpatient, outpatient and prescription drug claims for a sample of employees, dependents and retirees in the United States with primary or Medicare coverage through privately insured fee‐for‐service, point‐of‐service or capitated health plans.^16^ The present study was exempted from University of Minnesota Institutional Review Board approval because we analysed deidentified data.

### 2.2 Study population and design

We employed an active comparator, new user design^17^ and a target trial emulation approach.^14^, ^15^ We followed the TARGET (Transparent Reporting of Observational Studies Emulating a Target Trial) guidelines^15^ and in Table 1 compared the design of the target trial we sought to emulate with our study. Briefly, the treatment strategies included two GLP‐1 RAs indicated for obesity: for semaglutide, Wegovy® (maintenance dose 2.4 mg); for liraglutide, Saxenda® (maintenance dose 3.0 mg). Patients were eligible if they were ≥18 and ≤99 years old and did not have evidence of prior diabetes (type 1 or 2), stroke, myocardial infarction or heart failure. Additionally, we required a 6‐month “enrolment window”^18^ between insurance enrolment and index date. Follow‐up began on the date of first GLP‐1 RA prescription fill and concluded at the time of insurance disenrollment or administrative censoring on 31 December 2023. The causal contrast of interest was the intention‐to‐treat effect comparing use of semaglutide with use of liraglutide. Hazard ratios (HRs) were used to compare the effect of the treatment strategies on the incidence of type 2 diabetes and CVD.

**TABLE 1:** Target trial specification and emulation.

	Target trial specification	Target trial emulation using MarketScan data
Eligibility criteria	Age ≥18 and ≤99 years at enrolment BMI ≥ 30 kg/m^2^ or BMI ≥ 27 kg/m^2^ with a weight‐related comorbidity No history of type 1 or type 2 diabetes, confirmed by HbA1C levels <6.5% No history of myocardial infarction, stroke or heart failure No contraindication to GLP‐1 RA use	Same as for the target trial, except Patients prescribed the drugs of interest were assumed to meet BMI eligibility requirements and have no contraindications to GLP‐1 RA use. Diabetes‐free status determined by evidence of diabetes‐related claims over ≥6 months of observation prior to index date; therefore, patients lacking 6 months of observation were also excluded.
Treatment strategies	Weekly semaglutide (2.4 mg) or daily liraglutide (3.0 mg)	Same as for the target trial
Assignment procedure	Random allocation	Observational data did not permit random allocation. However, treatment assignment was assumed to be equivalent to random allocation conditional on matching on age, sex, insurance enrolment date and index date, as well as regression adjustment for propensity score and age.
Follow‐up initiation	Date of treatment assignment	Date of first GLP‐1 RA prescription fill
Follow‐up conclusion	Study withdrawal or completion of 60 months of follow‐up	Time of insurance disenrollment or 31 December 2023
Outcome definition	Type 2 diabetes, CVD (stroke, myocardial infarction or heart failure hospitalization) or composite CVD (CVD, coronary revascularization or unstable angina)	Same as for the target trial
Causal contrast	Hazard ratios for intention‐to‐treat effect (participants evaluated according to their initial treatment assignment, irrespective of adherence)	Same as for the target trial
Data analysis	Cox proportional hazards regression	Same as for the target trial

A graphical representation of the study design according to the approach recommended by Schneeweiss et al.^19^ is depicted in Figure 1. We defined the index date as the first instance of a prescription fill for semaglutide or liraglutide, which we ascertained using National Drug Code (NDC) numbers. This approach allowed us to identify GLP‐1 RA use during the titration phase (during which dosing is incrementally adjusted upwards) while distinguishing the intended exposure from other brand names of semaglutide and liraglutide prescribed for diabetes. Eligibility was determined, treatment assignment was made, and follow‐up began on the index date.^20^ The index date was identified prior to application of the exclusion criteria.^19^ Because semaglutide received FDA approval for chronic weight management in 2021 and because we later matched semaglutide users and liraglutide users on index date (see Section 2.4), the index date of patients in the analytic sample ranged from 2021 to 2023.

**FIGURE 1:**
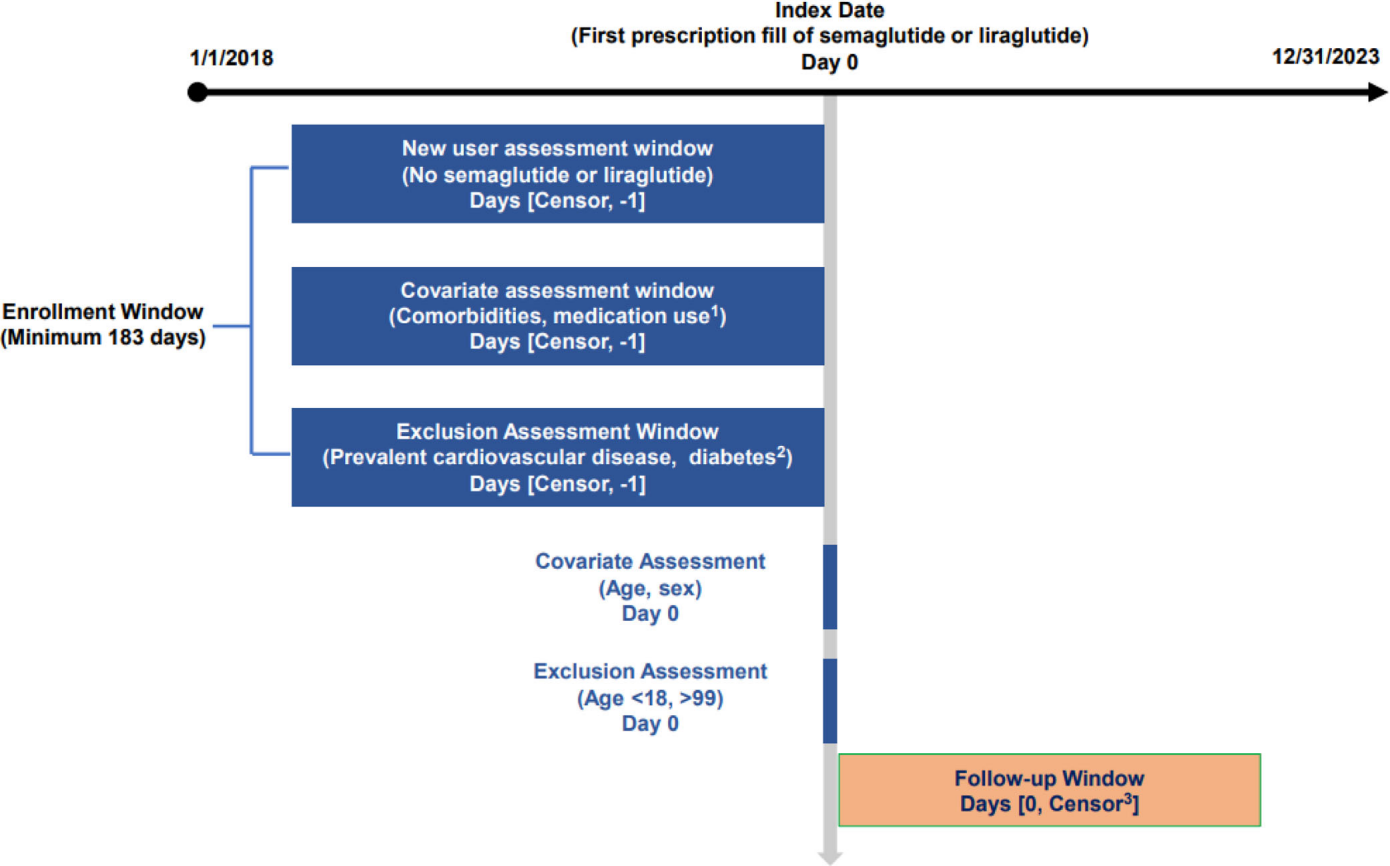
Graphical depiction of variable and follow‐up assessment by time, MarketScan 2018–2023. ^1^Conditions included hypertension, kidney disease, liver disease, depression, asthma, chronic obstructive pulmonary disease, hyperlipidaemia, obstructive sleep apnoea, obesity, tobacco use disorders, prediabetes and use of metformin, antihyperlipidemic medications and antihypertensive medications. ^2^Cardiovascular disease included myocardial infarction, heart failure and stroke; diabetes included type 1 and type 2 diabetes. ^3^Earliest of outcome of interest, insurance disenrollment (including death), 12/31/2023.

International Classification of Diseases, 10th Revision, Clinical Modification (ICD‐10‐CM) codes were used to identify the exclusion criteria of diabetes and CVD (Table S1). Although we required patients to have an enrolment window of at least 6 months (183 days) between insurance enrolment and the index date, information on GLP‐1 RA use, exclusion criteria and covariates was collected as early as 1 January 2018. This resulted in heterogeneous enrolment window lengths, but using this extra information (as opposed to ignoring it) has been shown to result in less biased estimates.^21^ In accordance with the intention‐to‐treat principle and as recommended in the pharmacoepidemiology methods literature,^22^ patients contributed follow‐up time based on the drug they were initially prescribed throughout the study, irrespective of adherence or switching. An additional GLP‐1 RA, tirzepatide, was approved for the treatment of obesity in November 2023 but was impractical to assess in the present study given that follow‐up concluded on 31 December 2023.

### 2.3 Outcomes

We investigated two primary endpoints: incident diabetes and incident CVD events. To capture diabetes diagnoses, we required one inpatient claim or two outpatient claims separated by more than 1 week but less than 1 year. CVD was defined as hospitalized myocardial infarction, stroke or heart failure. As a secondary endpoint, we analysed a composite CVD outcome, which further included unstable angina (from inpatient or outpatient ICD‐10‐CM codes) and coronary revascularization (from procedure codes). For the analysis of the composite CVD endpoint, enrolees with a history of unstable angina or coronary revascularization were excluded. Further details on outcome ascertainment algorithms are described in Table S1.

### 2.4 Statistical analysis

A nearest neighbour algorithm^23^ was used to match each liraglutide user with 1 semaglutide user based on age, sex, insurance enrolment date and index date, with a maximum distance of 3 years for age and 90 days for both enrolment date and index date. Characteristics of matched participants as of their index date were described according to treatment group using proportions and means. Standardized mean differences (SMDs) were calculated to assess between‐group differences. Rates of discontinuation were also calculated, with discontinuation defined as either a treatment switch or a >60‐day period between the end of the supply of one fill and the date of any subsequent fills.

We used Cox proportional hazards regression to calculate HRs and 95% confidence intervals (CIs), presenting results with liraglutide as the reference group. Censoring occurred at the time of insurance disenrollment (including death) or end of study follow‐up (31 December 2023). Models adjusted for age and a propensity score reflecting the probability of semaglutide *vs*. liraglutide prescription. The propensity score was fit using a logistic regression model where predictors included hypertension, kidney disease, liver disease, depression, asthma, chronic obstructive pulmonary disease (COPD), hyperlipidaemia, obstructive sleep apnoea, obesity, tobacco use disorders, prediabetes and use of metformin, antihyperlipidemic medications and antihypertensive medications. ICD‐10‐CM codes used to ascertain these variables are also listed in Table S1.

We tested for interactions by sex, given prior work suggesting sex differences in response to GLP‐1 RAs.^24^ The proportional hazards assumption was assessed by adding an interaction term between the exposure and follow‐up time. We also performed several sensitivity analyses: First, we extended the minimum enrolment window, matching individuals after excluding those who had <1 year (365 days) between insurance enrolment and the index date. Second, we implemented a more conservative approach to defining prevalent type 2 diabetes, excluding individuals with one outpatient type 2 diabetes code prior to matching (the definition of incident diabetes was not changed). Third, because there was a larger number of matching‐eligible semaglutide users than liraglutide users, we increased our sample by matching liraglutide users with up to two semaglutide users. In this analysis, models adjusted for sex in addition to age and propensity score. Fourth, using the entire sample eligible for matching, we compared semaglutide and liraglutide using stabilized inverse probability of treatment weighting. Propensity score weights were calculated using the same logistic regression model but also incorporated age and sex as predictors, and CIs were computed using a robust sandwich variance estimator. Finally, we conducted a per‐protocol analysis, censoring patients upon discontinuation or switching treatment. For all analyses, two‐sided *p*‐values < .05 were considered statistically significant. Analyses were performed using SAS version 9.4 (SAS Institute, Cary, NC).

## 3 RESULTS

Out of 33 920 450 unique individuals enrolled between 2021 and 2023, we identified 142 642 enrolees eligible for matching, including 103 533 semaglutide users and 39 109 liraglutide users. Our final matched sample included 57 456 individuals, including 28 728 semaglutide users and an equal number of liraglutide users (Figure 2). The mean age was 45 years, 83% were female, and 40% had hypertension. Although semaglutide and liraglutide users were not matched by the propensity score, the distributions of measured confounders were very similar between groups, with SMDs well below 0.1 in magnitude for all variables after matching (Table 2). We reported characteristics in all individuals eligible for matching in Table S2.

**FIGURE 2:**
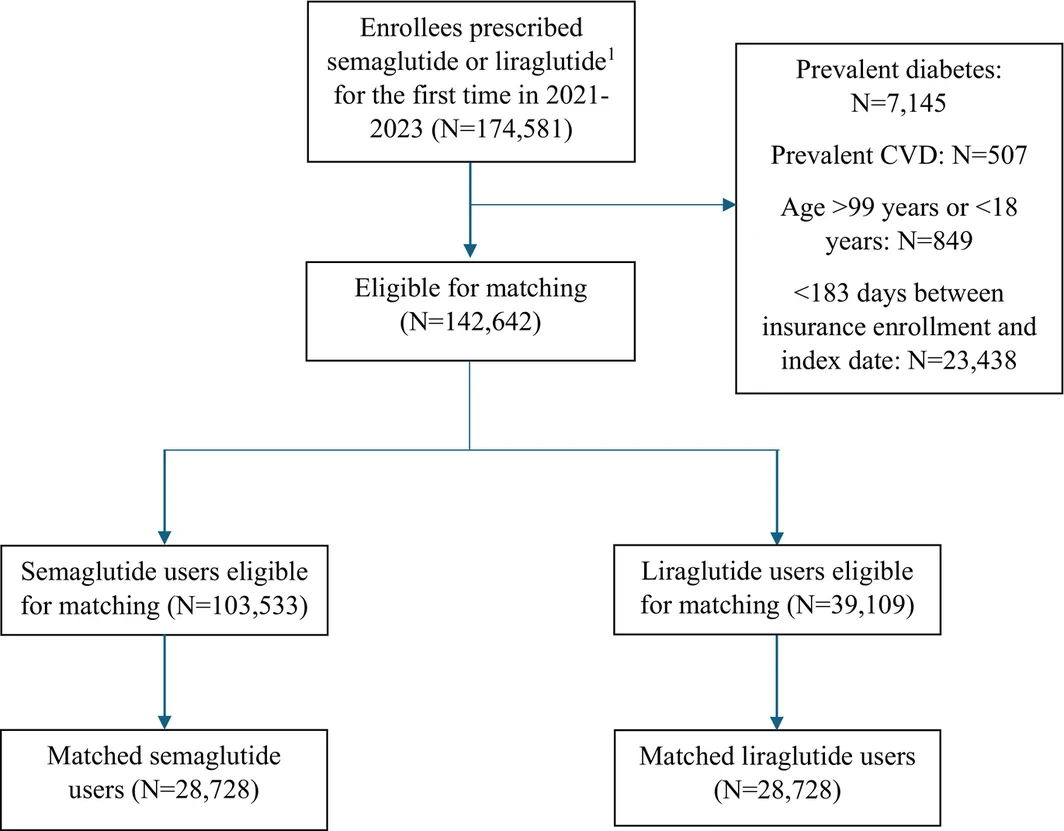
Subject flow chart (MarketScan enrolees 2021–2023, *N* = 33 920 450). ^1^The sample included only users of weekly semaglutide or daily liraglutide (brand names indicated for obesity management) who had not previously used any semaglutide or liraglutide drug.

**TABLE 2:** Matched patient characteristics.

	Semaglutide (*N* = 28 728)	Liraglutide (*N* = 28 728)	SMD
Age, years (SD)	45.1 (10.5)	45.0 (10.6)	0.00
Female sex (%)	83.3	83.3	0.00
Hypertension (%)	39.2	40.7	−0.03
Kidney disease (%)	1.9	1.8	0.01
Liver disease (%)	9.5	9.6	0.00
Depression	37.4	39.0	−0.03
Asthma (%)	14.2	14.4	−0.01
COPD (%)	7.9	8.0	0.00
Hyperlipidaemia (%)	39.9	39.8	0.00
Obstructive sleep apnoea (%)	18.6	19.2	−0.02
Obesity	75.6	76.6	−0.02
Tobacco use disorders	1.9	2.1	−0.01
Prediabetes (%)	25.2	24.9	0.01
Antihyperlipidemic medication use (%)	19.2	19.9	−0.02
Metformin use (%)	11.4	11.0	0.01
Any antihypertensive medication use (%)	46.6	47.5	−0.02
ACE inhibitors (%)	12.8	13.6	−0.02
Alpha‐blockers (%)	1.4	1.3	0.01
Angiotensin receptor blockers (%)	14.1	13.8	0.01
Beta‐blockers (%)	15.7	16.6	−0.02
Calcium channel blockers (%)	12.6	12.8	−0.01
Loop diuretics (%)	4.7	4.8	0.00
Potassium‐sparing diuretics (%)	8.7	8.2	0.02
Thiazide diuretics (%)	13.0	13.5	−0.02

*Note*: Patients were matched on age, sex, insurance enrolment date and GLP‐1 RA initiation date.

Abbreviations: ACE (angiotensin‐converting enzyme); COPD (chronic obstructive pulmonary disease); SD (standard deviation); SMD (standardized mean difference).

For the diabetes endpoint, median (Q1, Q3) follow‐up was 0.95 (0.48, 1.51) years. For CVD, median (Q1, Q3) follow‐up was 0.96 (0.49, 1.54) years. During follow‐up, 1104 individuals had an incident diabetes event and 57 had a CVD event. Of those who experienced a CVD event, 30 had incident myocardial infarction, 21 incident stroke and 6 incident heart failure. We identified 154 individuals who experienced a composite CVD event. We observed discontinuation in 53% of semaglutide users (including 6% who switched to liraglutide) and 79% of liraglutide users (including 33% who switched to semaglutide).

In the adjusted model, semaglutide use was associated with lower risk of incident diabetes (HR: 0.88 [95% CI: 0.78, 0.99]) compared with liraglutide use (Table 3). However, the proportional hazards assumption was violated in this analysis (*p* < .0001). Therefore, we stratified follow‐up at 6 months (0.5 years), at which point 42% of diabetes events had accrued. In the first 6 months of follow‐up, there was no relationship between semaglutide *vs*. liraglutide use and risk of diabetes (HR: 0.99 [95% CI: 0.82, 1.19]). Beyond the first 6 months after the index date, a significant association favouring semaglutide was present (HR: 0.80 [95% CI: 0.68, 0.94]).

**TABLE 3:** Association of semaglutide *vs.* liraglutide use and incident cardiometabolic outcomes, MarketScan 2021–2023.

Outcome	*N* events	Incidence rate^a^	Hazard ratio (95% CI)^b^	*p*‐value
Diabetes				
Semaglutide	513	17.6	0.88 (0.78, 0.99)	.03
Liraglutide	591	20.2	1 (Ref)	
Diabetes (<0.5 years)^c^				
Semaglutide	234	18.3	0.99 (0.82, 1.19)	.90
Liraglutide	233	18.5	1 (Ref)	
Diabetes (>0.5 years)				
Semaglutide	279	17.1	0.80 (0.68, 0.94)	<.01
Liraglutide	358	21.5	1 (Ref)	
CVD				
Semaglutide	31	1.1	1.25 (0.74, 2.11)	.40
Liraglutide	26	0.9	1 (Ref)	
Composite CVD				
Semaglutide	71	2.4	0.89 (0.65, 1.23)	.48
Liraglutide	83	2.8	1 (Ref)	

^a^ Per 1000 person‐years.

^b^ Models adjusted for age and a propensity score determined by hypertension, kidney disease, liver disease, depression, asthma, chronic obstructive pulmonary disease, hyperlipidaemia, obstructive sleep apnoea, obesity, tobacco use disorders, prediabetes and use of metformin, antihyperlipidemic medications and antihypertensive medications.

^c^ The diabetes analysis was stratified by follow‐up time due to violation of the proportional hazards assumption.

For both the CVD and composite CVD endpoints, a low number of events limited statistical precision. In each case, there was no evidence that the type of GLP‐1 RA affected risk of the outcome: The HR (95% CI) was 1.25 (0.74, 2.11) for CVD and 0.89 (0.65, 1.23) for composite CVD. We did not observe significant interactions by sex (data not shown).

In most sensitivity analyses performed, estimates were generally similar to those of the main analysis (Tables S3–S6). Specifically, the point estimate for incident diabetes was 0.88 for the analyses featuring a 1‐year minimum enrolment window, 0.83 using a more conservative exclusion of prevalent diabetes, 0.87 when we employed 1:2 matching of liraglutide users to semaglutide users and 0.90 when we accounted for confounding using inverse probability of treatment weighting. To facilitate comparison with the main results, the diabetes outcome was stratified by follow‐up time without regard to the *p*‐value of the test of proportional hazards, and the point estimates ranged from 0.94 to 0.99 during the first 6 months of follow‐up and 0.78 to 0.84 beyond that point for each of the sensitivity analyses. Estimates for the CVD and composite CVD endpoints were also comparable with those of the main analysis. Conversely, associations with diabetes were attenuated and became less precise in the per‐protocol analysis (Table S7).

## 4 DISCUSSION

In this target trial emulation using real‐world data, we performed a head‐to‐head comparison of the GLP‐1 RAs semaglutide and liraglutide (indicated for obesity management) among individuals without diabetes or prior CVD. We found that semaglutide use was associated with 12% lower risk of incident diabetes and that the association was more pronounced after the first 6 months of follow‐up. This finding is consistent with our hypothesis and was likely driven by the previously reported stronger effect of semaglutide on weight and HbA1C reduction.^10^, ^11^, ^12^ For CVD outcomes, conclusions were limited due to a low number of events resulting in imprecise estimates. Our results complement the findings of prior clinical trials as we report novel comparisons of two anti‐obesity drugs against each other, rather than placebo. Our study also has greater generalizability to the spectrum of individuals prescribed GLP‐1 RAs for obesity management than previous trials that recruited higher risk individuals with established CVD.

We found that use of semaglutide was associated with a 12% lower risk of developing diabetes compared with use of liraglutide and that this association was stronger during follow‐up time that occurred beyond the first 6 months after index date. Both semaglutide and liraglutide have been shown to reduce the incidence of diabetes compared with placebo.^6^, ^8^ Moreover, trials have randomized individuals to semaglutide or liraglutide therapy and compared effects on weight and biomarkers of hyperglycaemia, though they have not evaluated the risk of developing diabetes. The STEP 8 trial randomized 338 nondiabetic individuals with overweight or obesity to receive semaglutide or liraglutide and followed them for 68 weeks. Participants in the semaglutide arm had a greater reduction in HbA1C (difference: −0.2% [95% CI: −0.2, −0.1]) and fasting plasma glucose (difference: −3.9 mg/dL [95% CI: −7.2, −0.7]).^10^ The SUSTAIN 10 and PIONEER 4 trials, which compared varying doses and formulations of semaglutide and liraglutide in patients with diabetes, also reported stronger effects for semaglutide on weight and HbA1C after 30 and 26 weeks, respectively.^11^, ^12^ Results from these three trials align with our finding that use of semaglutide, *vs*. liraglutide, was associated with lower risk of developing diabetes. Additionally, these trials provide insight into our observation of an interaction with time. For each of the three trials, early study visits showed a stronger effect of liraglutide, but results favoured semaglutide by the end of follow‐up.^10^, ^11^, ^12^ The reason for this time interaction is unclear but may relate to the longer titration schedule of semaglutide (which can take up to 16 weeks compared with 4 weeks for liraglutide).^2^, ^3^, ^12^

We did not observe a difference in risk of developing CVD in the present head‐to‐head comparison of semaglutide and liraglutide. Clinical trials evaluating this comparison have yet to be performed, though trials of GLP‐1 RAs *vs*. placebo have shown benefit for CVD risk. In the SELECT trial, which enrolled individuals with obesity but without diabetes, semaglutide reduced the risk of major adverse cardiovascular events (MACE) by 20% compared with placebo over a 40‐month mean follow‐up (HR 95% CI: 0.72, 0.90).^7^ Among patients with diabetes enrolled in the LEADER trial, daily liraglutide (1.8 mg) lowered MACE by 13% over 45 months (HR 95% CI: 0.78, 0.97).^9^ Because CVD is the leading cause of death in the United States^25^ and obesity is a well‐established cardiovascular risk factor, even small effect sizes favouring one GLP‐1 RA over another in head‐to‐head comparison are important to detect. Given that our study was not powered to detect small effects (particularly for CVD endpoints), we recommend that future studies investigate this issue by analysing GLP‐1 RA use over greater follow‐up time than the average of 1 year that was available in the present study. In addition to short follow‐up, we also attribute our low event rate to the characteristics of our study sample, which was relatively young (mean age 45 years) and predominantly female (83%). These characteristics were likely influenced by our study design, which required that enrolees have no history of CVD or diabetes. Therefore, characteristics of our study sample differed markedly from those of prior cardiovascular outcomes trials^7^ but were comparable with those of other clinical trials and real‐world data studies with similar inclusion criteria.^10^, ^26^, ^27^

The high rates of discontinuation observed in our study were also commensurate with prior findings and may be attributable to product shortages, drug‐related adverse events or high out‐of‐pocket costs.^27^ This in turn led to imprecise findings in the per‐protocol analysis, to which we attribute the attenuated point estimates. One alternative conclusion from these results is that relative to liraglutide, semaglutide has stronger comparative effectiveness, but not efficacy. Notably, although power was limited to detect sex‐based interactions, no interaction was observed, and findings were relatively consistent in males and females in exploratory subgroup analysis.

### 4.1 Strengths and limitations

Our study had several strengths. First, we used an active comparator and a new user design and employed a target trial framework^14^ to compare two prominent GLP‐1 RAs using observational data. Second, the relatively large sample size afforded us by our data source allowed us to perform novel head‐to‐head comparisons in a study population more representative of the spectrum of GLP‐1 RA users without diabetes, where event rates were lower than those of prior clinical trials that primarily recruited individuals with established CVD. Third, we compared two drugs that had the same indication during the study period. In the two treatment groups, enrolee characteristics were highly similar after matching only on a limited set of variables including age and sex. This increases our confidence that unmeasured confounders were unlikely to have significantly biased the results of our analysis after propensity score adjustment.

We also had certain limitations. Because our study was observational, unmeasured confounding was still possible; we particularly note that we lacked measurements on and therefore could not account for differences in baseline characteristics such as weight and HbA1C, although our propensity score included variables that are strongly related to these characteristics. Additionally, some degree of misclassification of the exposures, outcomes, covariates and exclusion criteria was expected in this claims‐based analysis. To mitigate this issue, we used validated algorithms to ascertain these variables where possible. Also, as noted above, precision was poor for some analyses (particularly for the primary CVD outcome), and we may have been underpowered to detect sex interactions. One important difference between our analysis and the target trial we emulated relates to follow‐up time. Our analysis had a median follow‐up of approximately 1 year, which contributed to our low number of events in comparison with an ideal trial (e.g., we specified 5 years in Table 1). Finally, we note that results cannot be extrapolated to the uninsured US population. However, the costs of GLP‐1 RA use, which remain high, suggest their usage by uninsured individuals is not widespread.

### 4.2 Conclusion

Semaglutide use as compared with liraglutide use was associated with lower risk of incident diabetes in this real‐world, target trial emulation of GLP‐1 RAs prescribed to treat obesity. However, our head‐to‐head comparison did not reveal a difference in risk of CVD, though this may have been due to poor precision. Future research should compare semaglutide and liraglutide with tirzepatide and explore the long‐term cardiovascular effects of these drugs.

## AUTHOR CONTRIBUTIONS

E.J.C. and P.L.L. designed the research. E.J.C. conducted analyses and drafted the manuscript. W.W., F.L.N., R.F.W., S.K., L.S.C., J.F.F. and P.L.L. reviewed and edited the manuscript. All authors approved the final version of the manuscript. E.J.C. is the guarantor of this work and, as such, had full access to all the data in the study and takes responsibility for the integrity of the data and the accuracy of the data analysis.

## CONFLICT OF INTEREST STATEMENT

J.F.F. has received consulting support from AstaLynx Global and Prime Therapeutics. The other authors have no conflicts of interest to disclose.

## Supporting information


**Table S1:** Code algorithms used to identify study variables.
Table S2: Characteristics of patients eligible for matching.
Table S3: Results using expanded 1‐year minimum enrolment window.
Table S4: Results excluding individuals with any prior type 2 diabetes code.
Table S5: Results using 1:2 matching of liraglutide users to semaglutide users.
Table S6: Results using inverse probability of treatment weighting.
Table S7: Results following the per‐protocol approach^1^.

## Data Availability

Data may be obtained from a third party and are not publicly available: The licensing agreement through which we accessed the Merative MarketScan Commercial Claims and Medicare data prohibits us from sharing the data. However, other individuals or institutions may license the data through Merative: https://www.merative.com/documents/merative-marketscan-research-databases.
