# Supplementary material for: Semaglutide vs. liraglutide and incidence of diabetes and cardiovascular disease: A target trial emulation using real‐world data
Source: Br J Clin Pharmacol. 2026 Jul 16;92(11):3950–8. doi: 10.1002/bcp.70711 (PMC13714616; doi:10.1002/bcp.70711)
Supplement: Supplementary file 1 [file BCP-92-3950-s001.docx]

Supplemental Table 1: Code algorithms used to identify study variables

| Variable | Algorithm step | Codes used |
| --- | --- | --- |
| *Exclusion criteria and outcomes* |  |  |
| Type 1 Diabetes | One inpatient or one outpatient code in any position | ICD-10: E10 |
| Type 2 Diabetes^28^ | One inpatient or two outpatient codes (separated by ≥1 week and ≤1 year) in any position. | ICD-10: E11 |
| Myocardial infarction^29^ | One inpatient code in first position | ICD-10: I21, I22 |
| Stroke^30^ | One inpatient code in first position | ICD-10: I60, I61, I63, G46.3, G36.4, G46.5. G46.6, G46.7 |
| Heart failure^31^ | One inpatient code in first or second position | ICD-10: I50 |
| Unstable angina^32^ | One inpatient or outpatient code in any position | ICD-10: I24, I20.0, I25.110, I25.700, I25.710, I25.720, I25.730, I25.790 |
| Coronary revascularization^32^ | One inpatient or outpatient code in any position | CPT: 33510 – 33514, 33516 – 33519, 33521 – 33523, 33530, 33533 – 33536, 33572, 92920 – 92921, 92924 – 92925, 92928 – 92929, 92933 – 92934, 92937 – 92938, 92941, 92973, 92980 – 92982, 92984, 92995 – 92996  HCPCS: C9600 - C9606, G0290 - G0291  ICD-10-PCS: 037H34Z, 037H3DZ, 037H3ZZ, 037H44Z, 037H4DZ, 037H4ZZ, 037J34Z, 037J3DZ, 037J3ZZ, 037J44Z, 037J4DZ, 037J4ZZ, 037K34Z, 037K3DZ, 037K3ZZ, 037K44Z, 037K4DZ, 037K4ZZ, 037L34Z, 037L3DZ, 037L3ZZ, 037L44Z, 037L4DZ, 037L4ZZ, 037M34Z, 037M3DZ, 037M3ZZ, 037M44Z, 037M4DZ, 037M4ZZ, 037N34Z, 037N3DZ, 037N3ZZ, 037N44Z, 037N4DZ, 037N4ZZ, 037P34Z, 037P3DZ, 037P3ZZ, 037P44Z, 037P4DZ, 037P4ZZ, 037Q34Z, 037Q3DZ, 037Q3ZZ, 037Q44Z, 037Q4DZ, 037Q4ZZ, 03CH3ZZ, 03CJ3ZZ, 03CK3ZZ, 03CL3ZZ, 03CM3ZZ, 03CN3ZZ, 03CP3ZZ, 03CQ3ZZ, 057M3DZ, 057M4DZ, 057N3DZ, 057N4DZ, 057P3DZ, 057P4DZ, 057Q3DZ, 057Q4DZ, 057R3DZ, 057R4DZ, 057S3DZ, 057S4DZ, 057T3DZ, 057T4DZ, 037G34Z, 037G3DZ, 037G3ZZ, 037G44Z, 037G4DZ, 037G4ZZ, 057L3DZ, 057L4DZ, 02703ZZ, 02704ZZ, 02713ZZ, 02714ZZ, 02723ZZ, 02724ZZ, 02733ZZ, 02734ZZ, 02700ZZ, 02710ZZ, 02720ZZ, 02730ZZ, 02C00ZZ, 02C10ZZ, 02C20ZZ, 02C30ZZ, 3E07017, 3E070PZ, 3E07317, 3E073PZ, 02C03ZZ, 02C04ZZ, 02C13ZZ, 02C14ZZ, 02C23ZZ, 02C24ZZ, 02C33ZZ, 02C34ZZ, 021K0Z8, 021K0Z9, 021K0ZC, 021K0ZW, 021K4Z8, 021K4Z9, 021K4ZC, 021K4ZW, 021L0Z8, 021L0Z9, 021L0ZC, 021L4Z8, 021L4Z9, 021K0Z5, 021L0Z5, 021K4Z5, 021L4Z5, 02QA4ZZ, 02QB4ZZ, 02QC4ZZ, 02QA3ZZ, 02QB3ZZ, 02QC3ZZ, 0210344, 02103D4, 0210444, 02104D4, 0211344, 02113D4, 0211444, 02114D4, 0212344, 02123D4, 0212444, 02124D4, 0213344, 02133D4, 0213444, 02134D4, 02QA0ZZ, 02QB0ZZ, 02QC0ZZ |
| *Comorbidities* |  |  |
| Hypertension^33^ | One inpatient or outpatient code in any position | ICD-10: I10, I11, I12, I13, I15 |
| Kidney disease^33^ | One inpatient or outpatient code in any position | ICD-10: N18, N19, I12.0, I13.1, N03.2, N03.3, N03.4, N03.5, N03.6, N03.7, N05.2, N05.3, N05.4, N05.5, N05.6, N05.7, N25.0, Z49.0, Z49.1, Z49.2, Z94.0, Z99.2 |
| Liver disease | One inpatient or outpatient code in any position | ICD-10: B18, K73, K74, K70.0, K70.1, K70.2, K70.3, K70.4, K70.9, K71.1, K71.3, K71.5, K71.7, K72.1, K72.9, K76.0, K76.2, K76.3, K76.4, K76.5, K76.6, K76.7, K76.8, K76.9, Z94.4, I85.0, I85.9, I86.4, I98.2 |
| Depression^33^ | One inpatient or outpatient code in any position | ICD-10: F32, F33, F20.4, F31.3, F31.4, F31.5, F34.1, F41.2, F43.2 |
| Asthma^33^ | One inpatient or outpatient code in any position | ICD-10: J45.20, J45.21, J45.22, J45.30, J45.31, J45.32, J45.40, J45.41, J45.42, J45.50, J45.51, J45.52, J45.901, J45.902, J45.909, J45.990, J45.991, J45.998 |
| Chronic obstructive pulmonary disease | One inpatient or outpatient code in any position | ICD-10: J40, J41, J42, J43, J44, J47 |
| Hyperlipidemia | One inpatient or outpatient code in any position | ICD-10: E78.0-E78.5 |
| Obstructive sleep apnea | One inpatient or outpatient code in any position | ICD-10: G47.33 |
| Obesity | One inpatient or outpatient code in any position | ICD-10: E66.0, E66.1, E66.2, E66.8, E66.9, E88.82, Z68.3, Z68.4, Z68.55, Z68.56 |
| Tobacco use disorders | One inpatient or outpatient code in any position | ICD-10: F17.2, O99.33, T65.211A, T65.212A, T65.213A, T65.214A, T65.221A, T65.222A, T65.223A, T65.224A, T65.291A, T65.292A, T65.293A, T65.294A, Z72.0 |
| Prediabetes | One inpatient or outpatient code in any position | ICD-10: R73 |

Supplemental Table 2: Characteristics of patients eligible for matching

|  | Semaglutide  (N=103,533) | Liraglutide  (N=39,109) | SMD^1^ |
| --- | --- | --- | --- |
| Age, years (SD) | 45.6 (10.6) | 44.9 (10.6) | 0.07 |
| Female sex (%) | 79.6 | 84.0 | -0.12 |
| Hypertension (%) | 39.3 | 41.0 | -0.03 |
| Kidney disease (%) | 1.9 | 1.8 | 0.01 |
| Liver disease (%) | 9.4 | 9.4 | 0.00 |
| Depression | 35.0 | 39.3 | -0.09 |
| Asthma (%) | 13.7 | 14.6 | -0.03 |
| COPD (%) | 8.1 | 8.1 | 0.00 |
| Hyperlipidemia (%) | 39.2 | 39.7 | -0.01 |
| Obstructive sleep apnea (%) | 18.3 | 19.3 | -0.03 |
| Obesity | 68.7 | 78.8 | -0.23 |
| Tobacco use disorders | 1.9 | 2.1 | -0.02 |
| Prediabetes (%) | 23.4 | 25.3 | -0.04 |
| Antihyperlipidemic medication use (%) | 21.0 | 19.4 | 0.04 |
| Metformin use (%) | 10.5 | 11.3 | -0.03 |
| Any antihypertensive medication use (%) | 47.4 | 47.3 | 0.00 |
| ACE inhibitors (%) | 13.5 | 13.6 | -0.00 |
| Alpha-blockers (%) | 1.4 | 1.3 | 0.01 |
| Angiotensin receptor blockers (%) | 15.0 | 13.5 | 0.04 |
| Beta-blockers (%) | 16.3 | 16.4 | -0.00 |
| Calcium channel blockers (%) | 13.6 | 12.6 | 0.03 |
| Loop diuretics (%) | 4.7 | 4.9 | -0.01 |
| Potassium-sparing diuretics (%) | 8.2 | 8.1 | 0.00 |
| Thiazide diuretics (%) | 13.1 | 13.5 | -0.01 |

^1^Abbreviations: SMD (standardized mean difference); SD (standard deviation); COPD (chronic obstructive pulmonary disease); ACE (angiotensin-converting enzyme)

Supplemental Table 3: Results using expanded 1-year minimum enrollment window

| Outcome | N events | Incidence Rate^1^ | Hazard ratio (95% CI)^2^ | P-value |
| --- | --- | --- | --- | --- |
| Diabetes  Semaglutide  Liraglutide | 439  505 | 16.6  18.9 | 0.88 (0.78, 1.00)  1 (Ref) | .05 |
| Diabetes (<0.5 years)^3^  Semaglutide  Liraglutide | 192  193 | 16.7  17.0 | 0.98 (0.80, 1.20)  1 (Ref) | .85 |
| Diabetes (>0.5 years)  Semaglutide  Liraglutide | 247  312 | 16.6  20.4 | 0.82 (0.69, 0.97)  1 (Ref) | .02 |
| CVD  Semaglutide  Liraglutide | 30  26 | 1.1  1.0 | 1.22 (0.72, 2.06)  1 (Ref) | .47 |
| Composite CVD  Semaglutide  Liraglutide | 63  76 | 2.4  2.8 | 0.86 (0.62, 1.20)  1 (Ref) | .38 |

^1^Per 1000 person-years

^2^Models adjusted for age and a propensity score determined by hypertension, kidney disease, liver disease, depression, asthma, chronic obstructive pulmonary disease, hyperlipidemia, obstructive sleep apnea, obesity, tobacco use disorders, prediabetes and use of metformin, antihyperlipidemic medications and antihypertensive medications

^3^Diabetes analyses were stratified by time without performing a test for interaction to match the results of the main analysis

Supplemental Table 4: Results excluding individuals with any prior type 2 diabetes code

| Outcome | N events | Incidence Rate^1^ | Hazard ratio (95% CI)^2^ | P-value |
| --- | --- | --- | --- | --- |
| Diabetes  Semaglutide  Liraglutide | 355  439 | 12.7  15.6 | 0.83 (0.72, 0.95)  1 (Ref) | <.01 |
| Diabetes (<0.5 years)^3^  Semaglutide  Liraglutide | 98  103 | 8.0  8.5 | 0.94 (0.71, 1.24)  1 (Ref) | .66 |
| Diabetes (>0.5 years)  Semaglutide  Liraglutide | 257  336 | 16.4  20.9 | 0.79 (0.67, 0.93)  1 (Ref) | <.01 |
| CVD  Semaglutide  Liraglutide | 29  23 | 1.0  0.8 | 1.29 (0.75, 2.23)  1 (Ref) | .36 |
| Composite CVD  Semaglutide  Liraglutide | 69  71 | 2.5  2.5 | 0.99 (0.71, 1.38)  1 (Ref) | .94 |

^1^Per 1000 person-years

^2^Models adjusted for age and a propensity score determined by hypertension, kidney disease, liver disease, depression, asthma, chronic obstructive pulmonary disease, hyperlipidemia, obstructive sleep apnea, obesity, tobacco use disorders, prediabetes and use of metformin, antihyperlipidemic medications and antihypertensive medications

^3^Diabetes analyses were stratified by time without performing a test for interaction to match the results of the main analysis

Supplemental Table 5: Results using 1:2 matching of liraglutide users to semaglutide users

| Outcome | N events | Incidence Rate^1^ | Hazard ratio (95% CI)^2^ | P-value |
| --- | --- | --- | --- | --- |
| Diabetes  Semaglutide  Liraglutide | 763  591 | 17.5  20.2 | 0.87 (0.78, 0.97)  1 (Ref) | <.01 |
| Diabetes (<0.5 years)^3^  Semaglutide  Liraglutide | 379  233 | 18.2  18.5 | 0.99 (0.84, 1.16)  1 (Ref) | .86 |
| Diabetes (>0.5 years)  Semaglutide  Liraglutide | 384  358 | 16.8  21.5 | 0.78 (0.68, 0.90)  1 (Ref) | <.01 |
| CVD  Semaglutide  Liraglutide | 52  26 | 1.2  0.9 | 1.40 (0.88, 2.25)  1 (Ref) | .16 |
| Composite CVD  Semaglutide  Liraglutide | 116  83 | 2.6  2.8 | 0.97 (0.73, 1.28)  1 (Ref) | .81 |

^1^Per 1000 person-years

^2^Models adjusted for age and a propensity score determined by hypertension, kidney disease, liver disease, depression, asthma, chronic obstructive pulmonary disease, hyperlipidemia, obstructive sleep apnea, obesity, tobacco use disorders, prediabetes and use of metformin, antihyperlipidemic medications and antihypertensive medications

^3^Diabetes analyses were stratified by time without performing a test for interaction to match the results of the main analysis

Supplemental Table 6: Results using inverse probability of treatment weighting

| Outcome | N events^1^ | Incidence Rate^2^ | Hazard ratio (95% CI)^3^ | P-value |
| --- | --- | --- | --- | --- |
| Diabetes  Semaglutide  Liraglutide | 1320  900 | 17.1  19.6 | 0.90 (0.82, 0.99)  1 (Ref) | .02 |
| Diabetes (<0.5 years)^4^  Semaglutide  Liraglutide | 807  321 | 17.7  18.3 | 0.98 (0.86, 1.11)  1 (Ref) | .71 |
| Diabetes (>0.5 years)  Semaglutide  Liraglutide | 513  579 | 16.1  20.4 | 0.84 (0.74, 0.95)  1 (Ref) | <.01 |
| CVD  Semaglutide  Liraglutide | 77  44 | 1.0  0.9 | 1.07 (0.70, 1.63)  1 (Ref) | .77 |
| Composite CVD  Semaglutide  Liraglutide | 199  129 | 2.6  2.8 | 0.92 (0.72, 1.17)  1 (Ref) | .47 |

^1^Values for N events and person-years are based on the unweighted sample

^2^Per 1000 person-years

^3^Models adjusted for age and a propensity score determined by hypertension, kidney disease, liver disease, depression, asthma, chronic obstructive pulmonary disease, hyperlipidemia, obstructive sleep apnea, obesity, tobacco use disorders, prediabetes and use of metformin, antihyperlipidemic medication and antihypertensive medications

^4^Diabetes analyses were stratified by time without performing a test for interaction to match the results of the main analysis

Supplemental Table 7: Results following the per-protocol approach^1^

| Outcome | N events | Incidence Rate^2^ | Hazard ratio (95% CI)^3^ | P-value |
| --- | --- | --- | --- | --- |
| Diabetes  Semaglutide  Liraglutide | 213  166 | 14.5  17.2 | 0.98 (0.79, 1.20)  1 (Ref) | .81 |
| Diabetes (<0.5 years)^4^  Semaglutide  Liraglutide | 169  149 | 18.6  19.9 | 0.96 (0.77, 1.20)  1 (Ref) | .41 |
| Diabetes (>0.5 years)  Semaglutide  Liraglutide | 44  17 | 7.8  8.0 | 1.09 (0.62, 1.92)  1 (Ref) | .78 |
| CVD  Semaglutide  Liraglutide | 9  3 | 0.6  0.3 | 2.22 (0.59, 8.40)  1 (Ref) | .24 |
| Composite CVD  Semaglutide  Liraglutide | 24  21 | 1.6  2.2 | 0.86 (0.48, 1.57)  1 (Ref) | .63 |

^1^Patients were censored upon treatment discontinuation or switching

^2^Per 1000 person-years

^3^Models adjusted for age and a propensity score determined by hypertension, kidney disease, liver disease, depression, asthma, chronic obstructive pulmonary disease, hyperlipidemia, obstructive sleep apnea, obesity, tobacco use disorders, prediabetes and use of metformin, antihyperlipidemic medication and antihypertensive medications

^4^Diabetes analyses were stratified by time without performing a test for interaction to match the results of the main analysis
